# GM-CSF derived from the inflammatory microenvironment potentially enhanced PD-L1 expression on tumor-associated macrophages in human breast cancer

**DOI:** 10.1038/s41598-022-16080-y

**Published:** 2022-07-14

**Authors:** Kimihiro Yonemitsu, Cheng Pan, Yukio Fujiwara, Yuko Miyasato, Takuya Shiota, Hiromu Yano, Seiji Hosaka, Koji Tamada, Yutaka Yamamoto, Yoshihiro Komohara

**Affiliations:** 1 Department of Cell Pathology, Graduate School of Medical Sciences, Kumamoto University, Honjo 1-1-1, Chuouku, Kumamoto 860-8556 Japan; 2 Department of Immunology, Yamaguchi University Graduate School of Medicine, Yamaguchi, Japan; 3 Department of Breast and Endocrine Surgery, Kumamoto University Hospital, Kumamoto, Japan; 4 Center for Metabolic Regulation of Healthy Aging, Kumamoto University, Kumamoto, Japan

**Keywords:** Cancer, Immunology

## Abstract

Ever since immune checkpoint inhibitors have been approved for anti-cancer therapy in several cancers, including triple-negative breast cancer, the significance of programmed death-1 ligand 1 (PD-L1) expression in the tumor immune microenvironment has been a topic of interest. In the present study, we investigated the detailed mechanisms of PD-L1 overexpression on tumor-associated macrophages (TAMs) in breast cancer. In in vitro culture studies using human monocyte-derived macrophages, lymphocytes, and breast cancer cell lines, PD-L1 overexpression on macrophages was induced by the conditioned medium (CM) of activated lymphocytes, but not that of cancer cells. Granulocyte–macrophage colony-stimulating factor (GM-CSF) derived from activated lymphocytes was found to be involved in PD-L1 overexpression, in addition to interferon (IFN)-γ, via STAT3 pathway activation. Macrophages suppressed lymphocyte activation, and this inhibition was impaired by PD-1 blocking. The CM of activated lymphocytes also induced the overexpression of PD-L2, but GM-CSF did not affect PD-L2 expression. In the murine E0771 breast cancer model, anti-GM-CSF therapy did not affect PD-L1 expression on TAMs, and the mechanisms of PD-L1 expression on TAMs might differ between humans and mice. However, not only PD-L1, but also PD-L2 was overexpressed on TAMs in the E0771 tumor model, and their expression levels were significantly lower in the tumors in nude mice than in wild-type mice. Anti-PD-L1 antibody and anti-PD-L2 antibody synergistically inhibited E0771 tumor development. In conclusion, PD-L1 and PD-L2 were overexpressed on TAMs, and they potentially contributed to immunosuppression. The GM-CSF-STAT3 pathway is thought to represent a new mechanism of PD-L1 overexpression on TAMs in human breast cancer microenvironment.

## Introduction

Breast cancer is the most common cancer in women, and its incidence has been increasing worldwide. Breast cancer is classified into four molecular subtypes depending on the expression of hormone receptors and human epidermal growth factor receptor 2^1,2^. Triple-negative breast cancer (TNBC) is clinically defined by the absence of hormone receptor expression and human epidermal growth factor receptor 2 overexpression^3,4^. Neoadjuvant chemotherapy has been used as the standard treatment for patients with advanced breast cancer. An immunotherapy targeting programmed death 1 ligand 1 (PD-L1) has been approved in several countries for PD-L1-positive TNBC, and many clinical trials using immune checkpoint inhibitors in combination with compound-based drugs are now ongoing around the world^5,6^. Mismatch repair deficiency and PD-L1 expression on immune cells have been shown to be biomarkers for immunotherapy in TNBCs, and the tumor mutation burden, tumor-infiltrating lymphocytes (TILs), and transcriptional signatures of immune cells have also been suggested to be potential biomarkers^7,8^. Thus, recent advances in immunotherapy for solid cancers, including breast cancer, have shown the significance of the tumor immune microenvironment (TIME) in immune suppression in cancer patients.

Tumor-associated macrophages (TAMs) and TILs are the main components of the TIME in breast cancer cases. Several studies of solid tumors, including breast cancer, have indicated that a high density of TAMs was linked to a high malignant potential and a worse clinical course^9,10^, whereas a high density of TILs was associated with longer breast cancer-specific survival^11–13^. A high density of TILs in the TIME significantly predicted a pathological complete response by neoadjuvant chemotherapy in breast cancer^14,15^. We previously demonstrated that a high number of infiltrating CD204-positive protumor TAMs was a predictive marker for a worse clinical course in breast cancer^16^. Protumor TAMs are known to secrete several kinds of protumor molecules, including epidermal growth factor receptor ligands, interleukin (IL)-1β, IL-6, prostaglandin E2, tumor necrosis factor-α, oncostatin M, and osteopontin^17^.

In breast cancer, recent studies have indicated that PD-L1 is mainly expressed on immune cells, especially on TAMs^18^. PD-L1 expression on cancer cells (cut-off value: 1%) and immune cells (cut-off value: 10%) were detected in 12% and 28% of breast cancer samples, respectively, and high PD-L1 expression in immune cells predicted a better clinical course^19^. It was also reported that PD-L1 expression was closely associated with the interferon signature, indicating that PD-L1 expression reflects the anti-cancer immune responses in breast cancer^20^. Therefore, we examined the mechanisms of PD-L1 overexpression on TAMs and the immunosuppressive functions of TAMs, and found that lymphocyte-derived factors, but not cancer cell-derived factors, induced PD-1 ligand overexpression. In addition, although it is well known that interferons (IFNs) secreted from activated lymphocytes increase the expression of PD-1 ligands, we found that granulocyte–macrophage colony-stimulating factor (GM-CSF) secreted from activated lymphocytes also induced the overexpression of PD-1 ligands on macrophages via STAT3-related signals.

## Results

### Activated lymphocyte-derived factors induced PD-L1 overexpression in the breast cancer microenvironment

**Figure 1:**
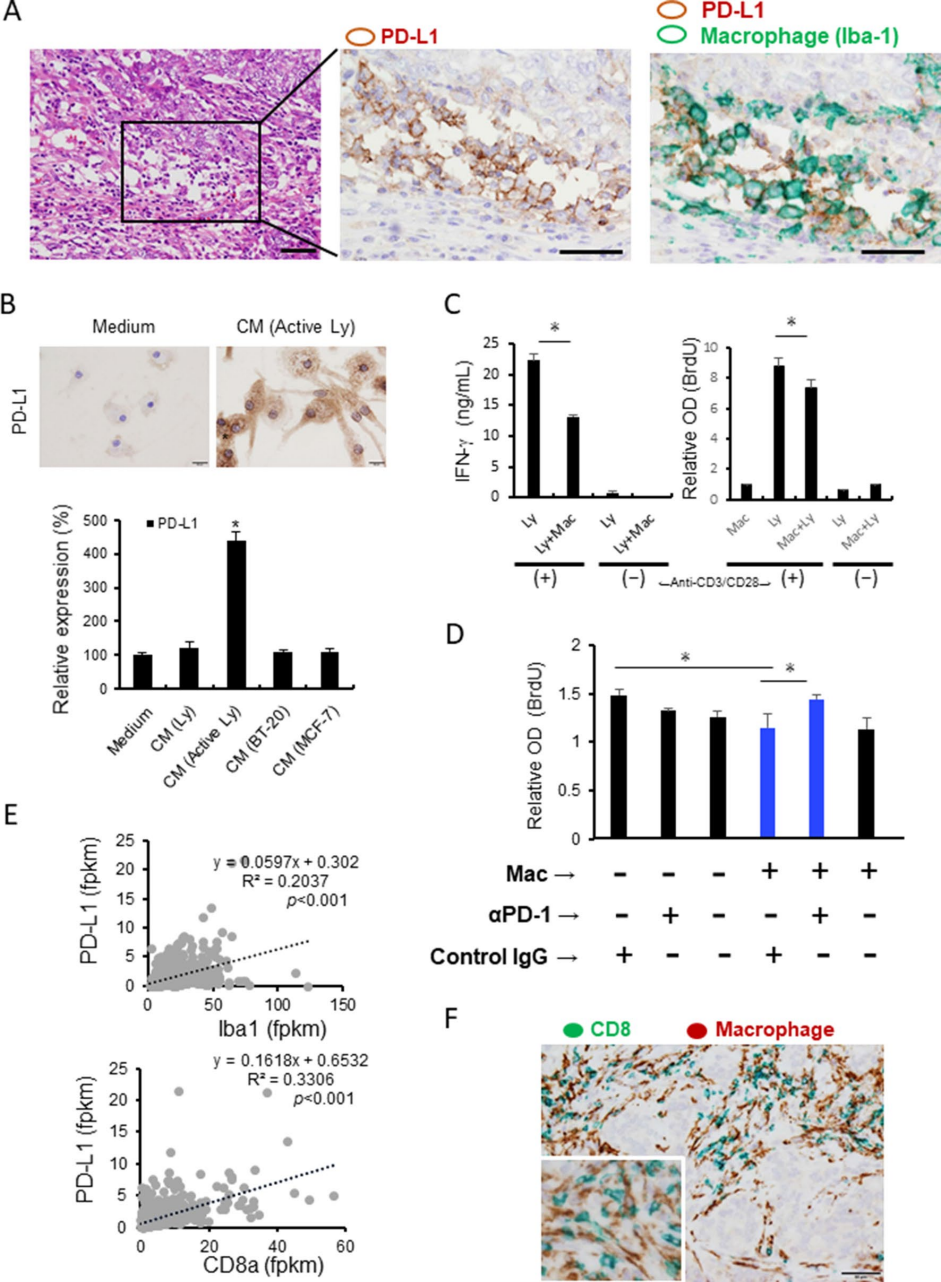
PD-L1 expression on macrophages. (**A**) Double-IHC of PD-L1 (brown) and Iba-1 (green; a pan-macrophage marker) in a breast cancer section is presented. Scale bar: 50 μm. (**B**) PD-L1 expression on HMDMs stimulated with the CMs from the resting lymphocytes, activated lymphocytes, BT-20 cell line, and MCF-7 cell line was evaluated by a cell-ELISA assay. PD-L1 staining on macrophages with or without CM stimulation is shown. (**C**) Autologous lymphocytes and macrophages were co-cultured in a culture plate coated with or without anti-CD3 and anti-CD28 antibodies. The IFN-γ concentration and BrdU incorporation were examined to evaluate lymphocyte activation. (**D**) Lymphocytes and macrophages were co-cultured in a culture plate coated with anti-CD3 and anti-CD28 antibodies, and anti-PD-1 antibody or control IgG was added. (**E**) The correlations between the PD-L1, Iba-1, and CD8a gene expression levels in breast cancer data from TCGA were tested by Spearman’s correlation test. (**F**) Double-IHC of Iba-1 (brown) and CD8 (green) in a breast cancer section is presented. Scale bar: 50 μm. *p-value < 0.05.

First, we examined whether macrophages expressed PD-L1 in breast cancer tissues by double-immunohistochemistry (IHC) of Iba1 (a pan-macrophage marker) and PD-L1. In a representative PD-L1-positive breast cancer case, many Iba1 and PD-L1 double-positive cells were observed infiltrating the cancer stroma (Fig. 1A), and this observation was consistent with a previous report^18^. Lymphocyte-derived factors are well known to be stimulators that induce PD-L1 expression; however, no studies on the association between breast cancer-derived factors and PD-L1 expression on human macrophages have been published. In the present study, human monocyte-derived macrophages (HMDMs) were stimulated with the conditioned medium (CM) of lymphocytes, the CM of lymphocytes activated with anti-CD3 and anti-CD28 antibodies, and the CM of breast cancer cell lines (BT-20 and MCF-7). A significant increase in PD-L1 expression was induced by the CM of activated lymphocytes; however, no change was observed in the HMDMs cultured with the CM of resting lymphocytes or breast cancer cell lines (Fig. 1B). Next, we examined whether macrophages stimulated with the CM of activated lymphocytes had immunosuppressive functions. Human autologous lymphocytes and HMDMs (pre-stimulated with the CM of activated lymphocytes) were co-cultured in a cell culture plate coated with anti-CD3 and anti-CD28 antibodies. As shown in Fig. 1C, the co-culture with HMDMs suppressed IFN-γ production and BrdU incorporation; this suppression of BrdU incorporation was abrogated by anti-PD-1 antibody (Fig. 1D). Based on The Cancer Genome Atlas (TCGA) database (https://www.proteinatlas.org/), PD-L1 expression is highly positively correlated with both CD8a and Iba-1 gene expression (Fig. 1E). In the same case shown in Fig. 1A, double-IHC showed that the CD8-positive lymphocytes and Iba1-positive macrophages were in direct contact (Fig. 1F).

### STAT1 and STAT3 signals were involved in PD-L1 overexpression on HMDMs

**Figure 2:**
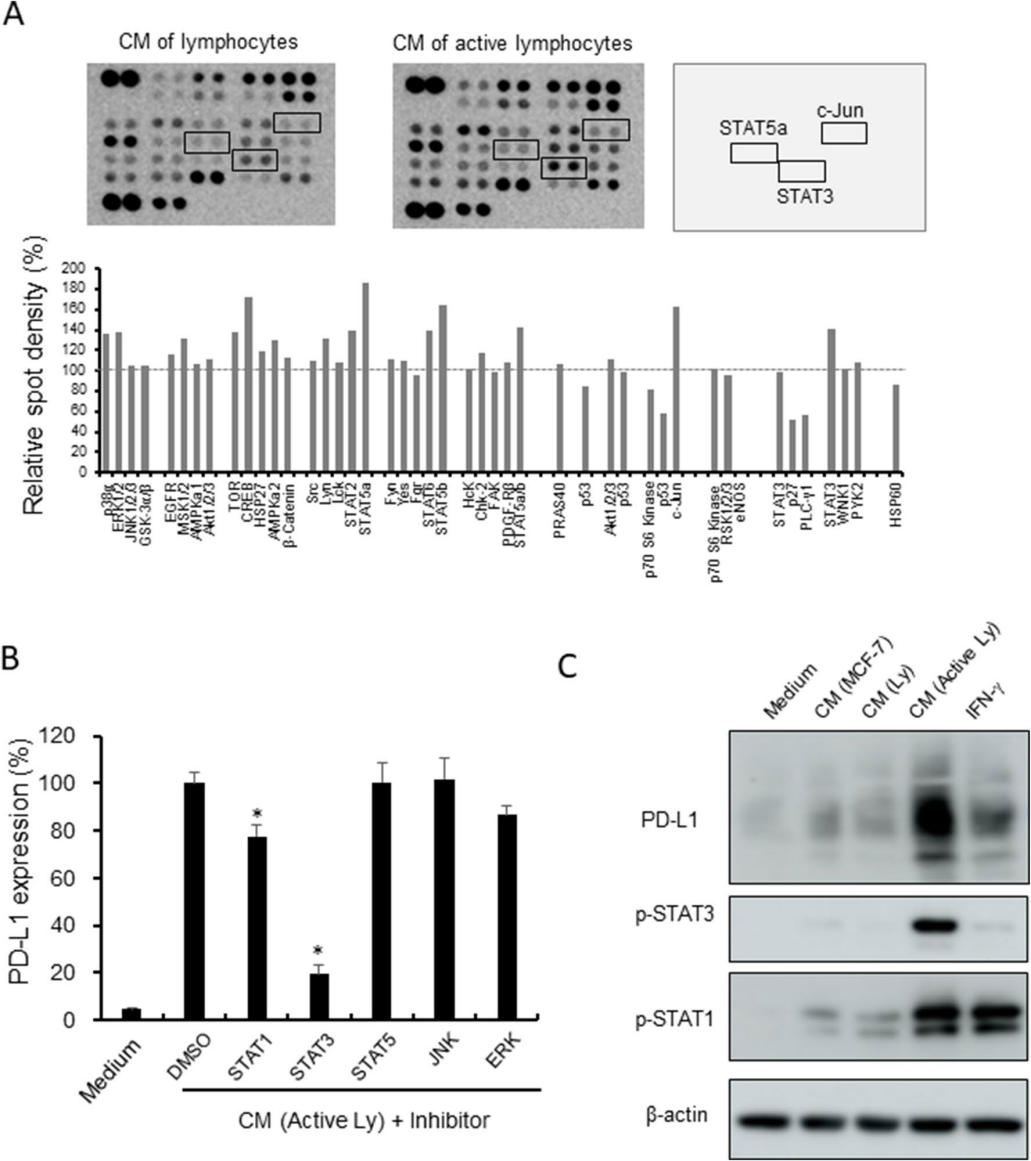
STAT1 and STAT3 activation in macrophages. (**A**) A phosphorylation kinase array was performed using HMDMs stimulated with the CM of resting lymphocytes and activated lymphocytes. Spot densities were evaluated by Image J software. Detailed molecules listed in a membrane was presented in supplemental Fig. 6A. (**B**) HMDMs were stimulated by the CM of activated lymphocytes (Active Ly) with inhibitors of STAT1, STAT3, STAT5, JNK, and ERK (10 nM) for 24 h, and PD-L1 expression was examined by a cell-ELISA assay (*n* = 3 to 5 each). (C) Western blot analysis of PD-L1, pSTAT1, pSTAT3, and β-actin was performed using the lysates of HMDMs stimulated with the CMs and IFN-γ for 24 h. *p-value < 0.05.

We next investigated the detail mechanisms of PD-L1 overexpression on HMDMs stimulated with the CM of activated lymphocytes. A phosphorylation kinase array analysis was then performed using the cell lysates of the HMDMs with the CM of lymphocytes or activated lymphocytes. The levels of some phosphorylation kinases were elevated; among them, we focused on STAT3, STAT5, and c-Jun (Fig. 2A), and we investigated the pathways that contribute to PD-L1 expression using inhibitors against these molecules. No direct inhibitor was available for c-Jun, so inhibitors of its upstream kinases, JNK and ERK, were used instead. A STAT1 inhibitor was also included since it has been reported that STAT1 induces PD-L1 expression in HMDMs ^21^. PD-L1 expression was strongly suppressed by the STAT1 and STAT3 inhibitors, with the STAT3 inhibitor showing a stronger inhibitory effect (79% reduction) than the STAT1 inhibitor (22% reduction; Fig. 2B). Western blot analysis was performed to examine whether STAT1 and STAT3 signals were activated by the CM of activated lymphocytes, and we found that the CM of activated lymphocytes induced the phosphorylation of both STAT1 and STAT3 (Fig. 2C).

### GM-CSF-related STAT3 activation enhanced IFN-γ-related PD-L1 overexpression on HMDMs

**Figure 3:**
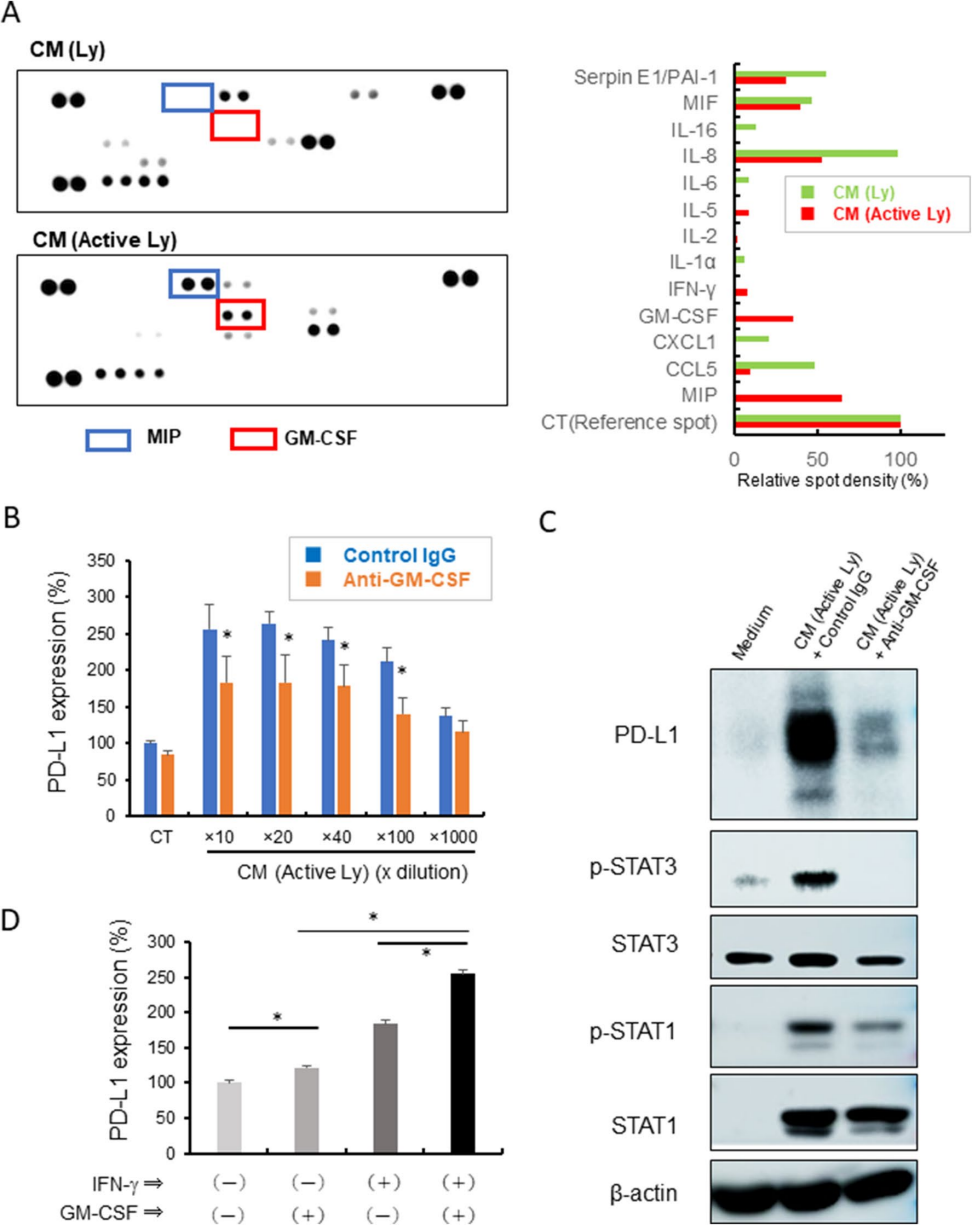
GM-CSF and PD-L1 expression. (**A**) A cytokine array was performed using the CMs of resting lymphocytes and activated lymphocytes. Spot densities were evaluated by Image J software. Detailed molecules listed in a membrane was presented in supplemental Fig. 6B. (**B**) HMDMs were stimulated by the CM of activated lymphocytes (Active Ly) with anti-GM-CSF antibody (20 μg/mL) or control IgG for 24 h, and PD-L1 expression was examined by a cell-ELISA assay (*n* = 3 to 5 each). *p-value < 0.05. (**C**) Western blot analysis of PD-L1, pSTAT1, pSTAT3, and β-actin was performed using the lysates of HMDMs stimulated with the CMs and antibodies for 24 h. (**D**) HMDMs were stimulated with IFN-γ (20 ng/mL) and GM-CSF (5 ng/mL) for 24 h, and PD-L1 expression was examined by a cell-ELISA assay (*n* = 4 each). *; *p* value = 0.0286.

Western blot analysis showed that the CM of activated lymphocytes, but not IFN-γ, induced the activation of STAT3 (Fig. 2C). This indicated that yet-unknown lymphocyte-derived factors are involved in PD-L1 overexpression on HMDMs via STAT3 activation. Using a cytokine array kit, we examined the kinds of cytokines that are present in the CM of activated lymphocytes. The levels of GM-CSF and macrophage migration inhibitory factor were significantly elevated in the CM of activated lymphocytes (Fig. 3A). Since it is well known that GM-CSF is linked to the STAT3 signaling pathway, we focused on GM-CSF. Antibody-mediated GM-CSF neutralization significantly suppressed PD-L1 overexpression and STAT3 activation (Fig. 3B and C). GM-CSF enhanced IFN-γ-induced PD-L1 expression on HMDMs (Fig. 3D). In a TCGA cohort of breast cancer cases, GM-CSF mRNA expression was seen in 375 (34.8%) of 1075 cases, and GM-CSF-positive cases showed higher gene expression levels of CD8a, Iba1, PD-L1, PD-L2, and lymphocyte activation-related genes such as IFN-γ and Granzyme B as compared to the levels in GM-CSF-negative cases (Supplemental Fig. 1A).

### PD-L2 expression was also observed on TAMs and HMDMs

**Figure 4:**
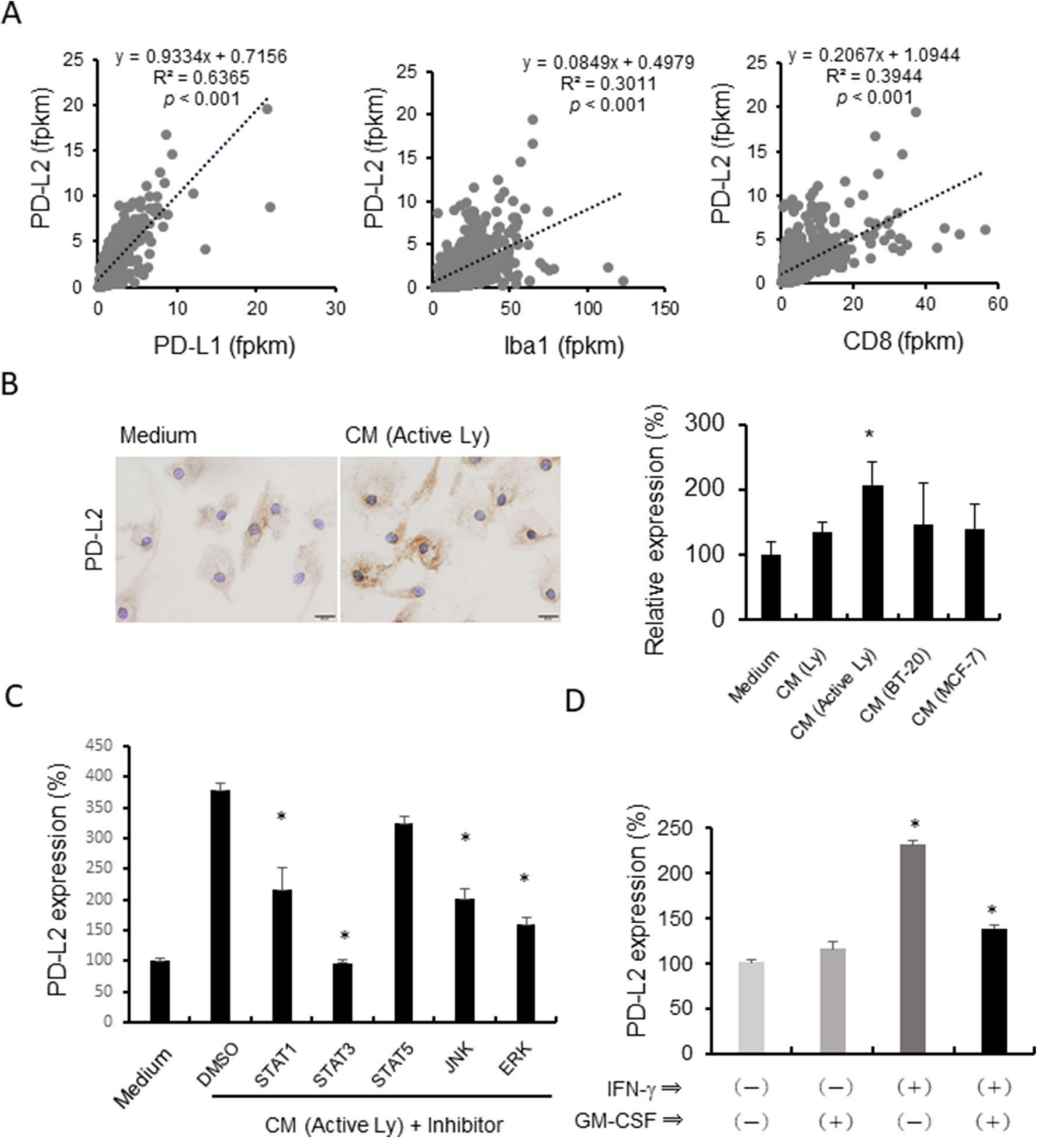
PD-L2 expression on macrophages. (**A**) The correlations between the PD-L1, PD-L2, Iba-1, and CD8a gene expression levels in breast cancer data from TCGA were examined by Spearman’s correlation test. (**B**) PD-L2 expression on HMDMs stimulated with the CMs of the resting lymphocytes, activated lymphocytes, BT-20 cell line, and MCF-7 cell line was evaluated by a cell-ELISA assay. PD-L2 staining on macrophages with or without CM stimulation is shown. (**C**) HMDMs were stimulated by the CM of activated lymphocytes (Active Ly) with inhibitors of STAT1, STAT3, STAT5, JNK, and ERK (10 nM) for 24 h, and PD-L2 expression was examined by a cell-ELISA assay (*n* = 3 to 5 each). (**D**) HMDMs were stimulated with IFN-γ (20 ng/mL) and GM-CSF (5 ng/mL) for 24 h, and PD-L2 expression was examined by a cell-ELISA assay (*n* = 4 each). *; *p* value = 0.0286.

A previous study showed that STAT3 activation was also linked to PD-L2 expression in HMDMs ^22^. Based on the TCGA database of breast cancer, the mRNA expression of PD-L2 is significantly associated with PD-L1 expression (Fig. 4A). The expression of both PD-L1 and PD-L2 was significantly correlated with CD8 and Iba1 gene expression. These findings suggested that PD-L2 was also overexpressed on TAMs and HMDMs. The in vitro studies using HMDMs indicated that the overexpression of PD-L2 was induced by the CM of activated lymphocytes as well as PD-L1 (Fig. 4B). PD-L2 overexpression was suppressed by inhibitors of STAT1, STAT3, JNK, and ERK (Fig. 4C); however, GM-CSF was not involved in PD-L2 expression (Fig. 4D).

### Inflammation-induced overexpression of PD-L1 and PD-L2 is a promising target for anti-cancer therapy

**Figure 5:**
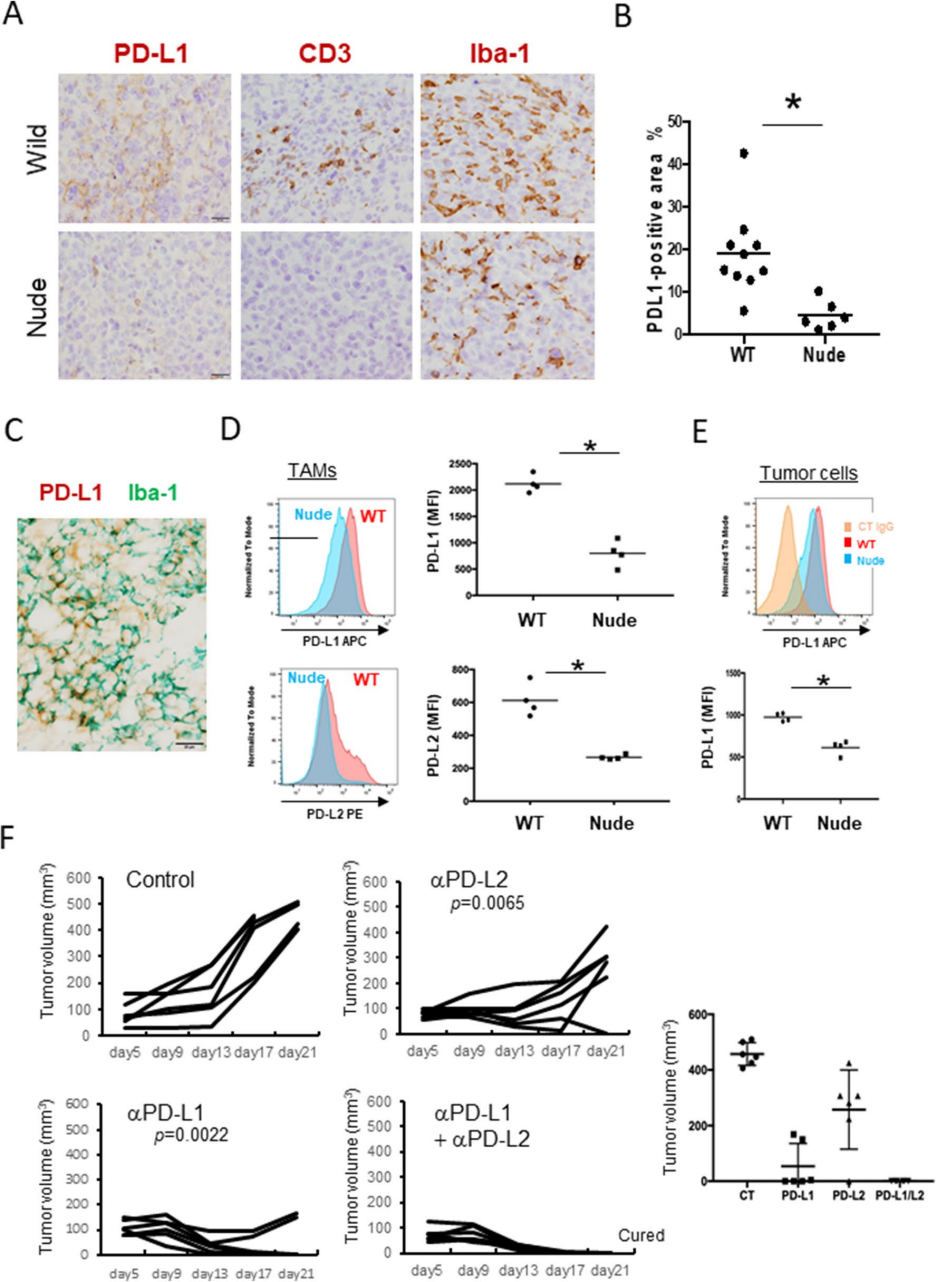
The murine E0771 breast cancer model. (**A**) E0771 cells were injected subcutaneously into the mice, and the resulting subcutaneous tumor nodules were resected and fixed in 4% paraformaldehyde. Paraffin sections were used for IHC of PD-L1, CD3, and Iba-1. (**B**) Positively stained areas of PD-L1 were evaluated by Image J software (*n* = 10 in wild-type (WT) mice, *n* = 6 in nude mice). (**C**) Double-IHC of PD-L1 (brown) and Iba-1 (green) was performed. Scale bar: 20 μm. (**D**) Tumor nodules from WT mice and nude mice were dissected (*n* = 4 in each group), and the PD-L1 and PD-L2 expression levels on CD11b^+^F4/80^+^ TAMs were analyzed by flow cytometry. The mean fluorescent intensity (MFI) of both proteins were evaluated. (**E**) PD-L1 on CD11b^-^ tumor cells was analyzed by flow cytometry (*n* = 4 in each group). (**F**) Anti-PD-L1 (αPD-L1), anti-PD-L2 (αPD-L1), and control hamster and rat IgG (100 μg/each) were injected intravenously at day 5 once, and tumor development was examined (*n* = 6 in each group). One of two representative experiments is presented.

Based on these observations, we hypothesized that inflammatory responses associated with TILs induce the overexpression of PD-1 ligands, which contributes to immune suppression. E0771 cells are a murine breast cancer cell line, and E0771 tumors are known to be an immunogenic “hot” tumor model. To examine the influence of TILs on the expression of PD-1 ligands in an in vivo model, E0771 cells were inoculated subcutaneously in wild-type mice and athymic nude mice. No TILs were observed in the tumor tissues that developed in the nude mice, whereas many TILs were found infiltrating the tumor tissues that developed in the wild-type mice (Fig. 5A). Higher PD-L1-positive signals were seen in the wild-type mice as compared to the nude mice (Fig. 5B), and double-IHC showed that PD-L1 was expressed on TAMs (Fig. 5C). Flow cytometry revealed that both PD-L1 and PD-L2 were overexpressed on CD11b^+^F4/80^+^ TAMs in the wild-type mice as compared to the nude mice (Fig. 5D). Tumor cells expressed PD-L1, and the expression level was lower in nude mice than in wild-type mice (Fig. 5E, Supplemental Fig. 2A); the mean fluorescent intensity of PD-L1 on tumor cells was half of that on the TAMs in the wild-type mice. No expression of PD-L2 was detected on the E0771 cells (Supplemental Fig. 2B). Next, anti-GM-CSF antibody was injected to neutralize the GM-CSF in the tumor microenvironment; however, the tumor growth and PD-L1 expression level remained unchanged (Supplemental Fig. 3). When anti-PD-L1 antibody and anti-PD-L2 antibody were injected to block the PD-1 signal, combination therapy with both antibodies completely suppressed tumor development (Fig. 5F).

## Discussion

In the present study, we showed by IHC that PD-L1 was overexpressed on TAMs, and by in vitro cell culture studies that infiltrating TILs affected the overexpression of PD-L1 on TAMs. The immunosuppressive function of PD-L1 on TAMs has been demonstrated in previous research using PD-L1-deficient mice^23,24^. In the present study, anti-PD-1 antibody increased the lymphocyte activation that was suppressed by macrophages. The in vitro studies using HMDMs demonstrated that soluble factors secreted by activated lymphocytes enhanced not only PD-L1, but also PD-L2; however, the mechanisms of PD-L2 overexpression appear to be different from those of PD-L1. A study using murine macrophages showed that PD-L1 expression was dependent on STAT1 and TLR4 signals, whereas PD-L2 expression was dependent on IL-4R and STAT6 signals^25^. The murine model using E0771 cells indicated that tumor cells expressed a low level of PD-L1, and were negative for PD-L2, whereas both PD-L1 and PD-L2 were expressed on TAMs. In a previous study using a MC38 murine tumor model, an elevated PD-L2 expression level was seen in TAMs treated with anti-PD-L1 antibody, and anti-PD-L2 antibody enhanced the anti-tumor immune responses induced by the anti-PD-L1 antibody^26^. Similar results were observed in the present study using the E0771 breast cancer model. These observations suggested that not only PD-L1, but also PD-L2 in the tumor microenvironment is a promising target for anti-tumor immunotherapy.

Many studies have demonstrated that gene amplification, 3’UTR disruption, and the activation of signaling molecules, such as STAT1/3, NF-kB, and HIF1a, are involved in PD-L1 overexpression in tumor cells^7^; however, there have been fewer studies on the PD-L1 overexpression on TAMs than that on tumor cells. PD-L1 expression on macrophages has been shown to be regulated by STAT1 and STAT3 signals^27^. We previously reported that lymphoma cell-derived IL-27 significantly enhanced PD-L1 expression on TAMs via the STAT3 pathway^22^. PD-L1 expression on macrophages is suggested to reflect the high IFN signature of immunologically “hot” tumor. In the present study, we showed that GM-CSF derived from activated lymphocytes enhanced IFN-induced PD-L1 expression on macrophages.

It is well known that lymphocyte-derived soluble factors, including IFN-γ, induce the overexpression of PD-L1 and PD-L2 on macrophages^28,29^. In the present study, lymphocyte-derived GM-CSF was found to enhance the IFN-γ-mediated PD-L1 overexpression via the activation of the STAT3 signaling pathway. PD-L2 expression on macrophages was increased by the CM of activated lymphocytes; however, the mechanisms of PD-L2 expression differed from those of PD-L1. GM-CSF is known to act as a survival and activating factor for myeloid cell maturation^30^. Although lymphocytes did not express receptors for GM-CSF, IL-2 induced GM-CSF expression in helper T cells via the STAT3 and STAT5 signaling pathways^31^. The levels of GM-CSF-producing CD4 and CD8 lymphocytes were shown to be increased in the blood and joints of patients with spondyloarthritis, and G protein-coupled receptor 65 was found to mediate GM-CSF production^32^. A deficiency in GM-CSF signaling suppressed the differentiation and maturation of alveolar macrophages, and contributed to the development of pulmonary alveolar proteinosis^33^. GM-CSF was the first cytokine shown to promote dendritic cell differentiation from monocytic lineage cells, and vaccination of GM-CSF gene-transfected cancer cells was reported to induce anti-cancer immune responses^34^. GM-CSF is produced not only by lymphoid cells, but also by endothelial cells and fibroblasts^34^. Ectopic GM-CSF expression in cancer cells has been reported in a small cell lung cancer cell line, and GM-CSF showed an anti-proliferative effect on cancer cells by arresting cells at the G0/G1 phases^35^. Chemo-resistant pancreatic cancer cells were shown to express GM-CSF, and blockade of GM-CSF improved the anti-cancer effect of chemotherapy by modulating the immunosuppressive tumor microenvironment^36^. Ectopic GM-CSF expression in lung cancer cells was enhanced by stimulation with chemotherapeutic drugs and induced PD-L1 overexpression in TAMs^37^. LLC murine lung cancer cell express expressed GM-CSF, and anti-GM-CSF therapy abrogated LLC tumor growth in vivo model by inhibiting TAM infiltration and differentiation. Murine breast cancer 4T1 cells were also shown to express GM-CSF, which promotes monocyte chemoattractant protein-1 (MCP-1) expression in macrophages; however, anti-GM-CSF therapy showed a limited effect on tumor growth^38^. Thus, multiple functions of GM-CSF in cancer biology have been reported, although few studies had investigated whether anti-GM-CSF therapy would be effective as a breast cancer therapy. To our knowledge, the present study is the first to describe the potential anti-tumor effect of anti-GM-CSF, which may be useful as a therapy for patients with breast cancer. However, anti-GM-CSF therapy showed a limited effect in the E0771 breast cancer model in the present study. IFN-γ, rather than GM-CSF, might be important for PD-L1 overexpression in TAMs in murine models.

In the present study, we focused on PD-L1 and PD-L2 as immunosuppressive molecules expressed on TAMs. We previously published two articles related to TAMs or lymphocytes in the tumor microenvironment in the same breast cancer cohort^16,39^. The two datasets on TAMs and lymphocytes were combined and re-analyzed, and we found that breast cancer cases with a high TIL density and high TAM density showed the worst clinical course, while cases with a high TIL density and low TAM density showed the best clinical course (Supplemental Fig. 4A). These observations indicated that TAMs potentially have an immunosuppressive function in breast cancer, and one possible mechanism is that PD-L1 overexpression is induced by GM-CSF/STAT3 signals (Supplemental Fig. 4B). Nevertheless, the growing amount of unprecedented data obtained from single-cell RNA sequencing has indicated that TAMs make up very heterogeneous cell populations with different functions in tumor immunity^40,41^.

In the present study, we could not confirm the GM-CSF expression in human breast cancer tissues, since no anti-GM-CSF antibody applicable to immunohistochemistry. However, there are some studies that demonstrated the positive correlation between the density of infiltrating lymphocytes and PD-L1 expression in breast cancer tissues^42,43^. There was also significant positive correlation between GM-CSF expression and lymphocyte-activation markers (Supplemental Fig. 1). Taken together with our observations in the present study, it was suggested that activating lymphocytes infiltrated in cancer stroma secreted GM-CSF which potentially affect PD-L1 overexpression on TAMs.

TAMs are known to secret protumor soluble factors, including IL-6, in several cancers. In cases with breast cancer, we previously demonstrated that osteopontin and heparin-binding epidermal growth factor-like growth factor (HB-EGF), in addition to IL-6, were involved in cancer cell growth^44^. We also examined which chemokines were potentially involved in TAM accumulation in the TIME. MCP-1 is the most well-known chemokine related to macrophage chemotaxis^45^; however, the level of CCL5 was increased by lymphocyte-derived factors, suggesting that CCL5, rather than MCP-1, is a critical chemotactic factor. Inhibitor of FROUNT, a coactivator for CCR2 and CCR5 signals, showed an inhibitory effect on E0771 tumor growth by blocking TAM infiltration^44^. Taken together, we propose the following mechanisms of TAM-related protumor signaling: (1) Cytotoxic signals from TILs induce chemokine production, such as CCL5, in breast cancer cells; (2) Infiltrated TAMs secret protumor soluble factors, including osteopontin, heparin-binding epidermal growth factor-like growth factor, and IL-6; and (3) TIL-derived factors, including IFN-γ and GM-CSF, induce PD-1 ligands, which in turn suppress the anti-cancer effects of TILs (Supplemental Fig. 5).

In conclusion, PD-L1 is preferentially overexpressed on TAMs, possibly due to the cell–cell interaction with TILs. Although IFN-γ is well known to be related to PD-L1 overexpression, GM-CSF derived from TILs was suggested to induce PD-L1 overexpression in a synergistic manner with IFN-γ via STAT3 signal activation. In the E0771 breast cancer model, TAMs expressed PD-L1 and PD-L2, and the expression levels of these molecules were suppressed in nude mice, suggesting that the inflammatory microenvironment induced the overexpression of PD-L1 and PD-L2. Anti-GM-CSF therapy showed a limited effect in the E0771 breast cancer model; however, combined anti-PD-L1 and PD-L2 therapy significantly suppressed cancer development. The present study results indicated a novel mechanism of PD-L1 overexpression on TAMs in the TIME.

## Materials and methods

### Immunohistochemistry

Paraffin Sections (3-μm thick) of breast cancer samples were used for immunohistochemical studies as described previously^46^. The following monoclonal antibodies were used as the primary antibodies: anti-Iba-1 antibody (NCNP27; WAKO, Tokyo, Japan) and anti-CD8 antibody (C8/144B; Nichirei, Tokyo, Japan). After the samples were reacted with these primary antibodies, they were incubated with horseradish peroxidase-labeled secondary anti-mouse antibody (Nichirei). The reaction was visualized using the diaminobenzidine system (Nichirei). No signal was observed when normal mouse immunoglobulin (Ig; DAKO, Glostrup, Denmark) was used. The DAKO automated system (Autostainer Link 48; DAKO) was used for the IHC analysis of human PD-L1 (clone 22C3; DAKO). For double-IHC, the sections were washed with citrate buffer (pH 2.2), then reacted with anti-Iba-1 or anti-CD8 antibody, and visualized with HistoGreen (Linaris, Heidelberg, Germany).

### Cell culture of macrophages

HMDMs were obtained from healthy donors in accordance with protocols approved by the Kumamoto University Hospital Review Board (No. 1169), and cultured as described previously^16^. In brief, monocytes were isolated using RosetteSep Human Monocyte Enrichment Cocktail (STEMCELL Technologies, Vancouver, Canada). Then, the cells were cultured in AIM-V medium (Thermo Fisher, Waltham, MA, USA) supplemented with macrophage-colony stimulating factor (100 ng/mL; WAKO) and 2% human serum for 7 days to induce monocyte differentiation into macrophages. Recombinant GM-CSF and IFN-γ were obtained from WAKO.

### Cell culture of lymphocytes

Human T-lymphocytes were isolated from healthy donors using RosetteSep Human T-cell Enrichment Cocktail (STEMCELL Technologies). Lymphocytes were cultured in a cell culture plate coated with anti-human CD3 antibody (OKT3; eBiosciences, San Diego, CA, USA) and human CD28 antibody (BioLegend, San Diego, CA, USA). The proliferation of lymphocytes was examined by the BrdU incorporation assay (Cell Proliferation ELISA kit, Roche, Basel, Switzerland). Anti-PD-1 antibody (clone EH12.2H7) and isotype-matched IgG were obtained from BioLegend.

### Cell lines

Human breast cancer cell lines, BT-20 and MCF-7, were purchased from the Japanese Collection of Research Bioresources Cell Bank (Osaka, Japan). The murine breast cancer cell line E0771 was obtained from CH3 BioSystem (Amherst, NY, USA). All cells were cultured in DMEM/F12 (WAKO) with 10% fetal bovine serum (Invitrogen).

### Cell enzyme-linked immunosorbent assay (ELISA)

HMDMs (2 × 10^4^ cells/well) were cultured with the CM of cancer cell lines at a CM:culture medium ratio of 1:4 for 1 day in a 96-well microplate. Following fixation with 1% paraformaldehyde for 10 min, the cells were reacted with biotin-labeled anti-PD-L1 antibody (clone 29E.2A3; BioLegend), anti-PD-L2 antibody (clone 24F.10C12; BioLegend), or isotype-matched control antibody (BioLegend). After the wells were washed with phosphate-buffered saline, horseradish peroxidase-labeled streptavidin (Nichirei) was added. Tetramethylbenzidine developing solution (BioLegend) was used to visualize the positive signals.

### Western blot analysis

#### Western blot analysis

After the protein concentration in the cell lysates was quantified using the bicinchoninic acid assay, equal amounts of protein were separated by electrophoresis and transferred onto a polyvinylidene fluoride membrane. The following antibodies were used for western blotting: anti-PD-L1 antibody (clone E1L3N), anti-STAT1 antibody (clone 42H3), anti-pSTAT1 antibody (clone D4A7), anti-STAT3 antibody (clone 124H6), and anti-pSTAT3 antibody (clone Y705; all from Cell Signaling Technology).

#### Flow cyt*ometry*

Tumor nodules were treated with Tumor & Tissue Dissociation Reagent (Becton Dickinson, Franklin Lake, NJ, USA). The resulting cell suspensions were treated with FcR-blocking reagent (BioLegend), then the cells were reacted with APC anti-mouse PD-L1 antibody (10F.9G2), PE anti-mouse PD-L2 antibody (TY25), PE/Cyanine7 anti-mouse F4/80 antibody (BM8), Violet 51 anti-mouse/human CD11b antibody (M1/70), or isotype-matched control IgGs (all from BioLegend). Dead cells were excluded by labeling with Fixable Viability Dye eFluor™ 780 (Invitrogen). The stained cell samples were analyzed on a FACSverse (Becton Dickinson) flow cytometer with FlowJo software (Becton Dickinson).

### Murine breast cancer model

E0771 cells (8 × 10^5^) in 50 μl of phosphate-buffered saline were inoculated subcutaneously into the left and right back of C57BL/6 J female mice (CLEA, Shizuoka, Japan). The hamster anti-mouse PD-L1 antibody (clone 10B5; 100 mg/mice) was established previously^39^, and control hamster IgG was obtained from Sigma (St. Louis, MO, USA). Anti-PD-L2 antibody (TY25) and rat isotype-matched control antibody were purchased from BioXel (New Haven, CT, USA). All animal procedures were planned according to the Animal Research: Reporting of In Vivo Experiments (ARRIVE) guidelines, and approved by the Animal Research Committee at Kumamoto University (#A2020-089).

### Statistics

Statistical analysis was carried out using GraphPad PRISM7 (https://www.graphpad.com) and JMP7 (SAS Institute, Chicago, IL, USA) software. Spearman’s correlation test and the Mann–Whitney U-test were used to test for correlations between two groups. Differences in mean values among multiple groups were analyzed by one-way analysis of variance. *P* values of < 0.05 were considered statistically significant. The cell count data of TAMs and TILs and the breast cancer-specific survival periods in a breast cancer cohort were obtained from two studies previously published by our groups^16,22^. The breast cancer-specific survival rates were compared between two groups using the log-rank test and Kaplan–Meier plots. All *p*-values are based on two-tailed statistical analyses, and *p*-values < 0.05 were considered to be statistically significant.

### Ethics

#### Approval for human experiments

The study design was approved by the Institutional Review Board of Kumamoto University (#2059) in accordance with the guidelines for Good Clinical Practice and the Declaration of Helsinki. HMDMs and lymphocytes were obtained from healthy donors in accordance with protocols approved by the Kumamoto University Hospital Review Board (No. 1169). The need for individual patient consent for inclusion in the study was waived by the Institutional Review Board of Kumamoto University (#2059) since the present study was a retrospective analysis using previously published data^16,22^; however, although all of the retrospective patient data were automatically included in the study, the patients were given the opportunity to refuse participation by opting out of the study.

#### Approval for animal experiments

All procedures were carried out in accordance with the relevant guidelines and regulations. All animal procedures were planned according to the Animal Research: Reporting of In Vivo Experiments (ARRIVE) guidelines, and were approved by the Animal Research Committee at Kumamoto University (#A2020-089).

## Supplementary Information

Supplementary Information.

## Data Availability

The datasets generated and/or analyzed in the current study are available from the corresponding author on reasonable request.
