# Supplementary material for: GM-CSF derived from the inflammatory microenvironment potentially enhanced PD-L1 expression on tumor-associated macrophages in human breast cancer
Source: Sci Rep. 2022 Jul 14;12:12007. doi: 10.1038/s41598-022-16080-y (PMC9283541; doi:10.1038/s41598-022-16080-y)
Supplement: Supplementary file 1 — Supplementary Information. [file 41598_2022_16080_MOESM1_ESM.pdf]

Supplemental figure 1

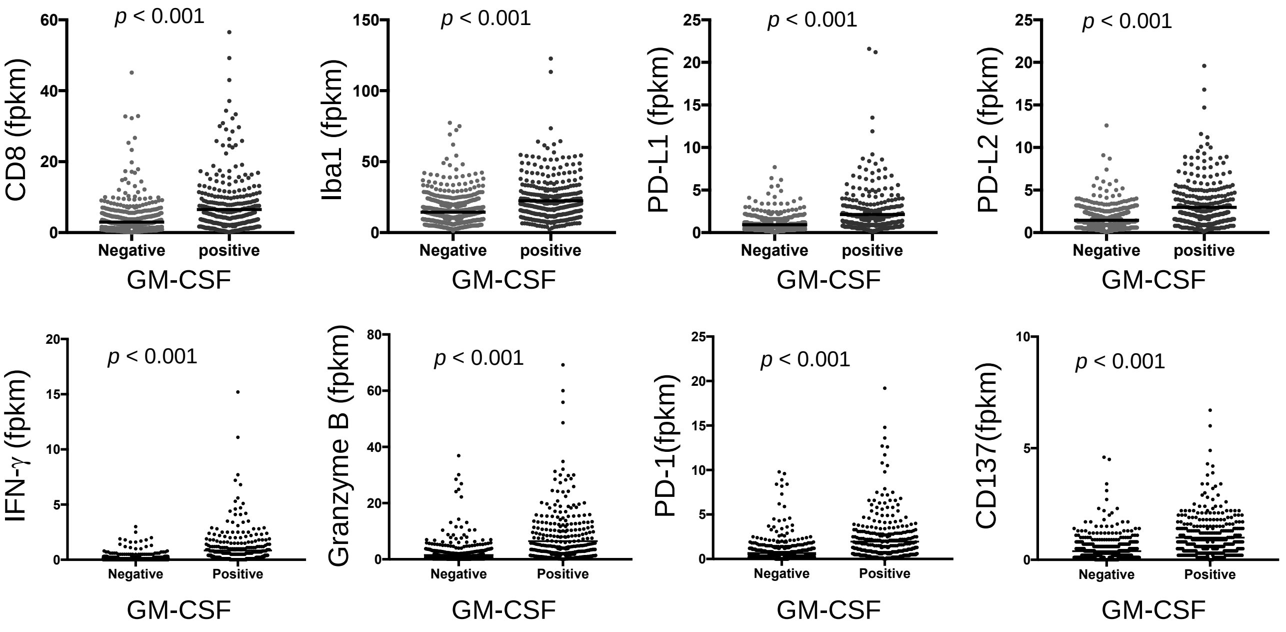

Supplemental Figure 1. Gene expression analysis in breast cancer cases from TCGA database. Gene expression levels of CD8, Iba1, PD-L1, PD-L2, IFN-γ, Granzyme B, PD-1, and CD137 in breast cancer were compared between the cases with and without GM-CSF expression. The Mann-Whitney *U*-test was performed.

Supplemental figure 2

A

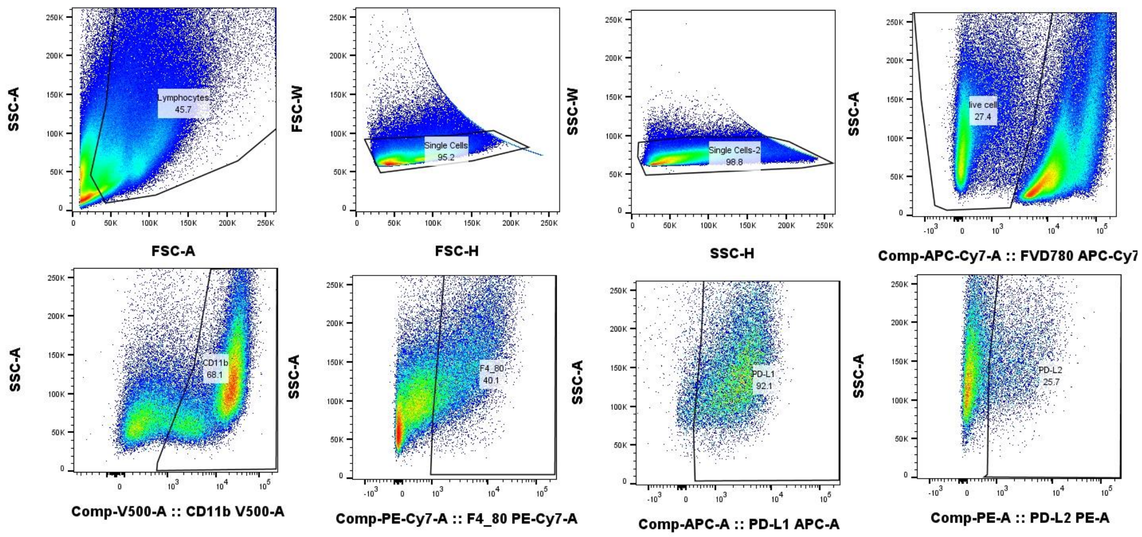

B

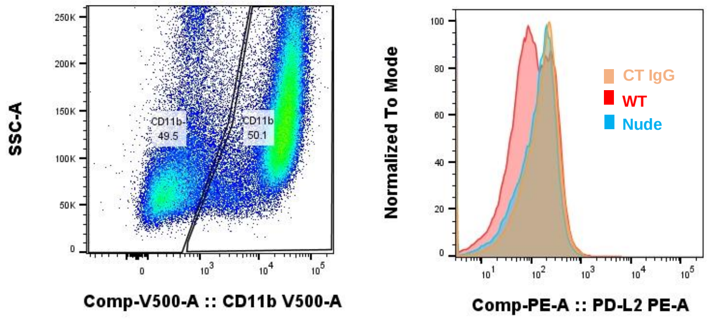

Supplemental Figure 2. Flow cytometry of an E0771 tumor sample. (A) Cell suspensions were stained with APC anti-mouse PD-L1 antibody, PE anti-mouse PD-L2 antibody, PE/Cyanine7 anti-mouse F4/80 antibody, Violet 510 anti-mouse/human CD11b antibody, or isotype-matched control IgGs. Dead cells were excluded by staining with Fixable Viability Dye eFluor™ 780. (B) The data of PD-L2 in the same experiment of figure 5E.

Supplemental figure 3

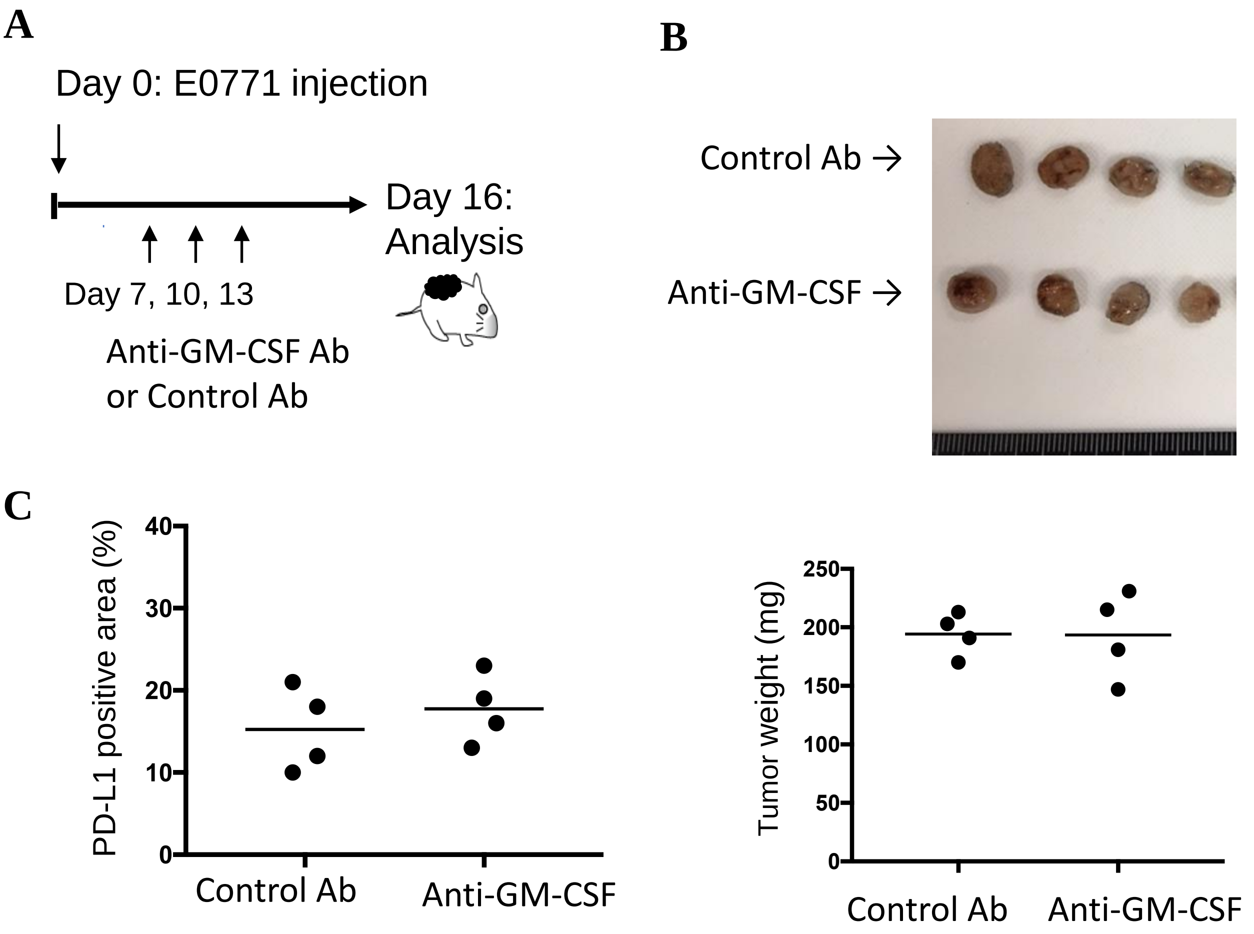

Supplemental figure 3. Effect of anti-GM-CSF antibody on the E0771 model. (A) The protocol of anti-GM-CSF antibody therapy. The tumor weight was evaluated and compared between the anti-GM-CSF -treated group and the control antibody-treated group antibody (100μg per mice/n=4 in each group). Anti-GM-CSF antibody (clone MP1-22E9) and isotype-matched control antibody were purchased from BioXel (New Haven, CT, USA). (B) Tumor weight was evaluated at day 16. (C) The PD-L1-positive area (%) was examined by Image J software.

Supplemental figure 4

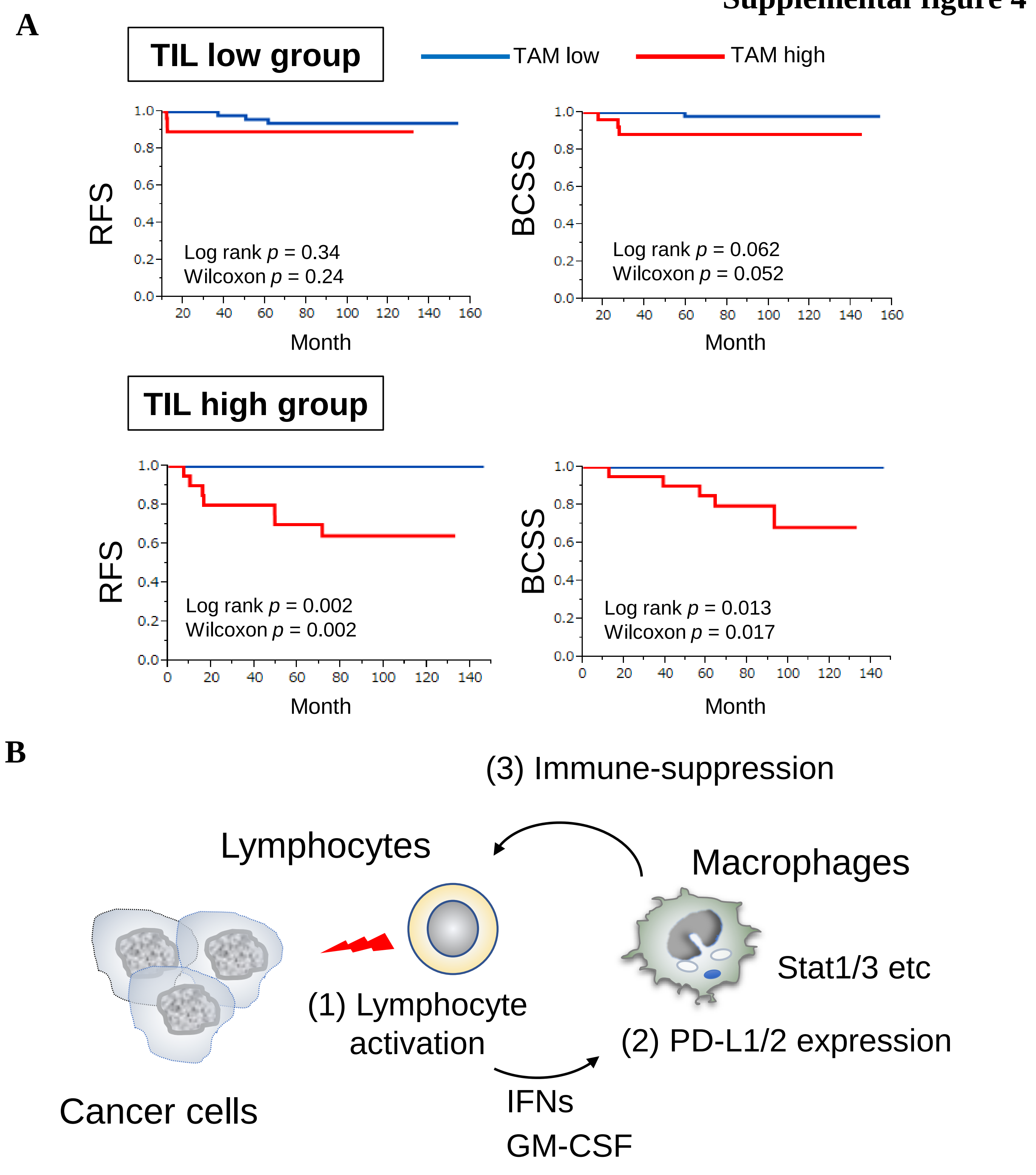

Supplemental figure 4. Clinical data of a breast cancer cohort and a suggested scheme of TAM-related immunosuppression. (A) Cases in a breast cancer cohort were divided into two groups according to the density of TILs (high and low), and the recurrence-free survival (RFS) and breast cancer-specific survival (BCSS) were compared between the two groups. (B) In the suggested scheme of TAM-related immunosuppression, IFNs and GM-CSF derived from activated lymphocytes induce PD-L1 and PD-L2 on TAMs. GM-CSF enhances IFN-induced PD-L1 overexpression on TAMs via STAT3 activation. Although the mechanisms of PD-L2 overexpression might be more complex, PD-L1 and PD-L2 expressed on TAMs are thought to be involved in the immunosuppression in the TIME.

Supplemental figure 5

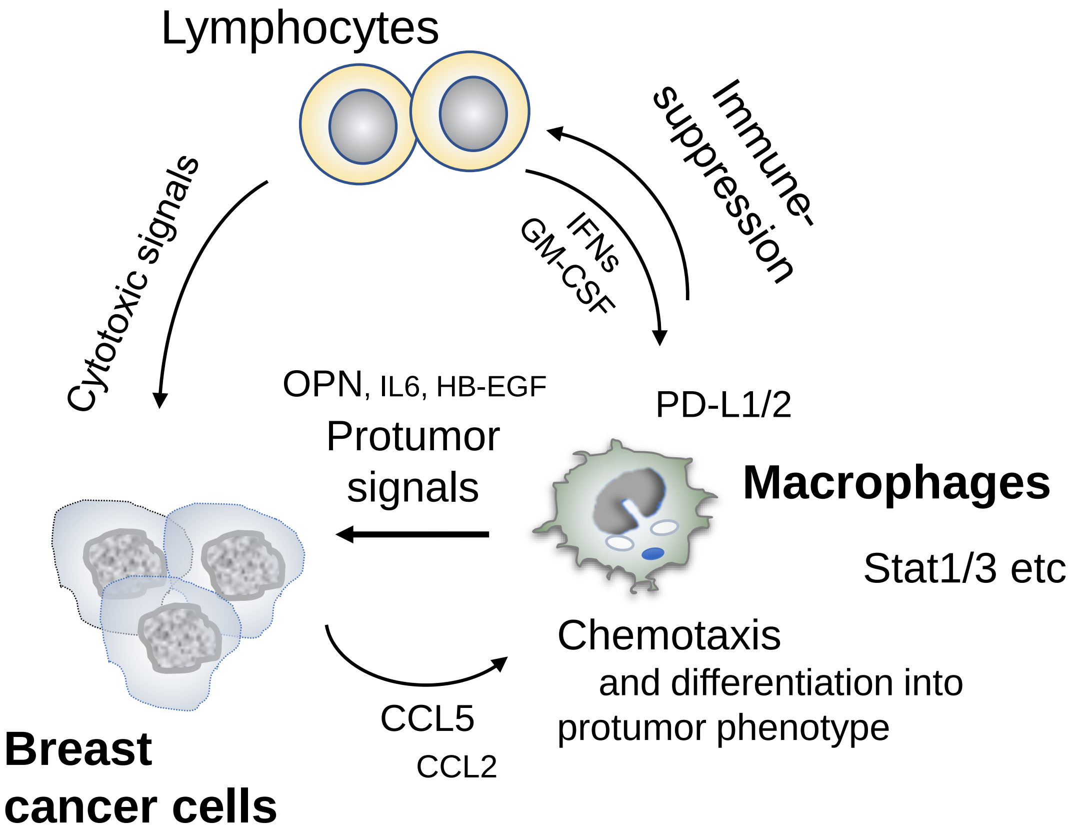

Supplemental Figure 5. Hypothetical functions of TAMs in the breast cancer TIME. Activated TILs stimulate the secretion of chemokines, such as CCL2 and CCL5, from breast cancer cells. CCL2 and CCL5 induce the chemotaxis of TAMs in the TIME, and cancer-derived factors enhance TAM activation into a protumor phenotype. TAMs secret protumor factors, including osteopontin (OPN), heparin-binding epidermal growth factor-like growth factor (HB-EGF), and IL-6. Activated TILs induced PD-L1 and PD-L2 overexpression on TAMs, which suppresses TIL activation. Thus, TAMs are involved in cancer progression via protumor and immunosuppressive functions.

Supplemental figure 6

A

	1, 2	3, 4	5,6	7,8	9,10
A	Reference Spot	p38α	ERK1/2	JNK 1/2/3	GSK-3α/β
B		EGF R	MSK1/2	AMPKα1	Akt 1/2/3
C	TOR	CREB	HSP27	AMPKα2	β-Catenin
D	Src	Lyn	Lck	STAT2	STAT5a
E	Fyn	Yes	Fgr	STAT6	STAT5b
F	Hck	Chk-2	FAK	PDGF Rβ	STAT5a/b
G	Reference Spot	PRAS40			PBS (Negative Control)

B

	1, 2	3, 4	5,6	7,8	9,10	11, 12	13, 14	15, 16	17, 18	19, 20
A	Reference Spot	CCL1/I-309	CCL2/MCP-1	MIP-1α/MIP-1β	CCL5/RANTES	CD40 Ligand/TNFSF5	Complement Component C5/C5a	CXCL1/GROα	CXCL10/IP-10	Reference Spot
B		CXCL11/I-TAC	CXCL12/SDF-1	G-CSF	GM-CSF	ICAM-1/CD54	IFN-γ	IL-1α/IL-1F1	IL-1β/IL-1F2	
C		IL-1ra/IL-1F3	IL-2	IL-4	IL-5	IL-6	IL-8	IL-10	IL-12 p70	
D		IL-13	IL-16	IL-17A	IL-17E	IL-18/IL-1F4	IL-21	IL-27	IL-32α	
E	Reference Spots	MIF	Serpin E1/PAI-1	TNF-α	TREM-1					Negative Control

Supplemental figure 6; Detailed columns of RTK array presented in figure 2A (A) and cytokine array presented in figure 3A (B).

Supplemental figure 7

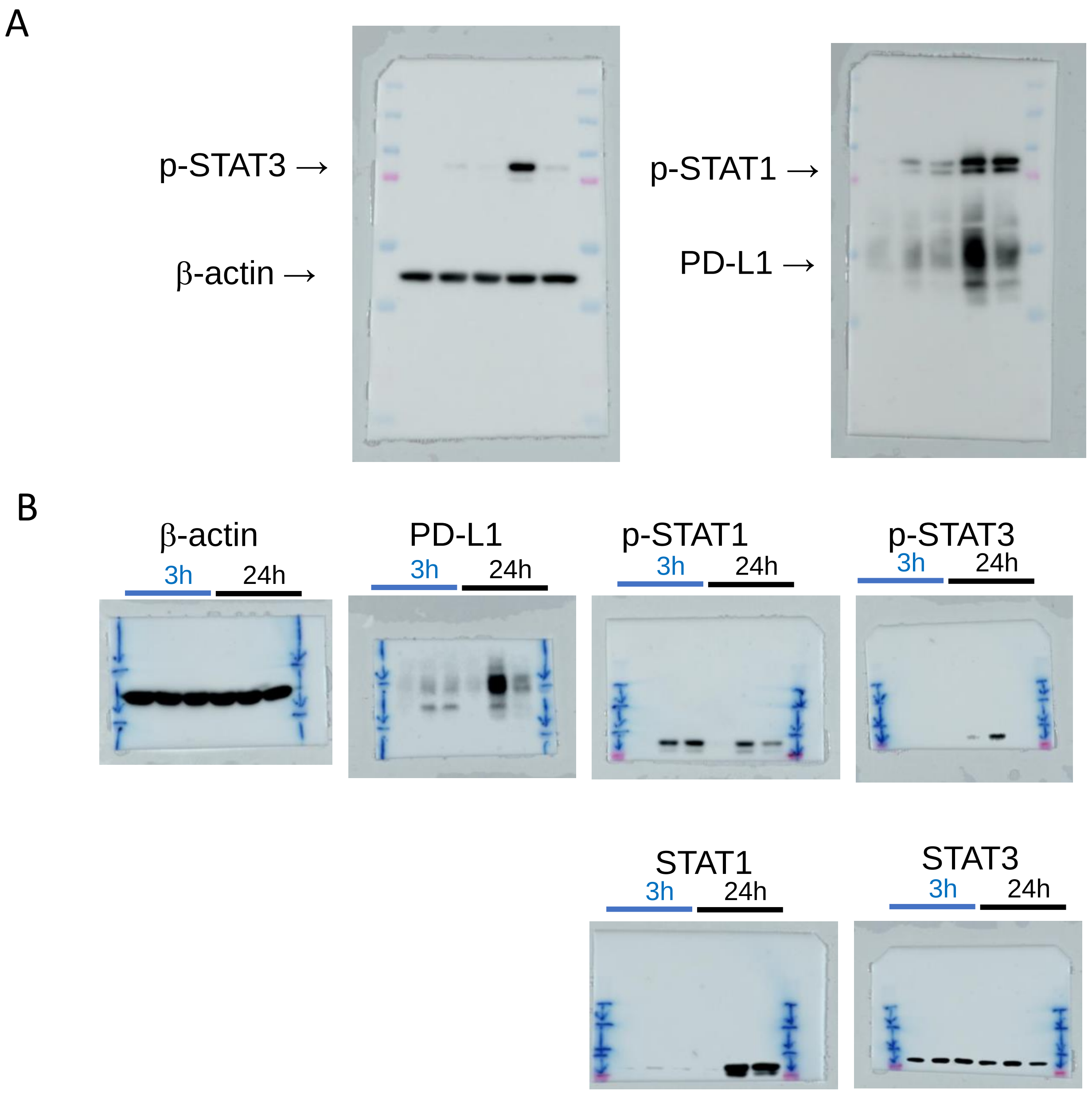

Supplemental Figure 7. Full-length gels or blots of figure 2C (A) and figure 3C (B) were presented.
